# Molecular characterization of AIFM2/FSP1 inhibition by iFSP1-like molecules

**DOI:** 10.1038/s41419-023-05787-z

**Published:** 2023-04-21

**Authors:** Thamara Nishida Xavier da Silva, Clemens Schulte, Ariane Nunes Alves, Hans Michael Maric, José Pedro Friedmann Angeli

**Affiliations:** 1 Rudolf Virchow Zentrum; Center for Integrative and Translational Bioimaging, University of Würzburg, Josef-Schneider-Str. 2, 97080 Würzburg, Germany; 2 Technische Universität Berlin; Institute of Chemistry, Straße des 17. Juni 124, 10623 Berlin, Germany

**Keywords:** Chemical libraries, Cell biology

## Abstract

Ferroptosis is a form of cell death characterized by phospholipid peroxidation, where numerous studies have suggested that the induction of ferroptosis is a therapeutic strategy to target therapy refractory cancer entities. Ferroptosis suppressor protein 1 (FSP1), an NAD(P)H-ubiquinone reductase, is a key determinant of ferroptosis vulnerability, and its pharmacological inhibition was shown to strongly sensitize cancer cells to ferroptosis. A first generation of FSP1 inhibitors, exemplified by the small molecule iFSP1, has been reported; however, the molecular mechanisms underlying inhibition have not been characterized in detail. In this study, we explore the species-specific inhibition of iFSP1 on the human isoform to gain insights into its mechanism of action. Using a combination of cellular, biochemical, and computational methods, we establish a critical contribution of a species-specific aromatic architecture that is essential for target engagement. The results described here provide valuable insights for the rational development of second-generation FSP1 inhibitors combined with a tracer for screening the druggable pocket. In addition, we pose a cautionary notice for using iFSP1 in animal models, specifically murine models.

## Introduction

Ferroptosis is a regulated cell death modality characterized by iron-dependent lipid peroxidation [1]. Ferroptosis has been implicated in a wide array of (patho)physiological conditions [2, 3], and more recently, numerous studies have proposed exploiting ferroptosis as a novel therapeutic strategy to induce cancer cell death in tumor entities lacking therapeutic options [4–8]. The selenoprotein glutathione peroxidase 4 (GPX4) has been identified as the major regulator of ferroptosis, having a crucial role in reducing peroxidized phospholipids and preventing cell death [9–11]. Subsequent studies have discovered alternative mechanisms that are able to suppress ferroptosis in the absence of GPX4 [12–16]. To this end, we demonstrated the importance of the flavoprotein ferroptosis suppressor protein 1 (FSP1), which is encoded by the gene apoptosis-inducing factor mitochondria-associated 2 (*AIFM2*). FSP1 suppresses ferroptosis by contributing to the regeneration of membrane-embedded antioxidants. Specifically, FSP1 restores ubiquinone to ubiquinol, using NAD(P)H as a cofactor, and thus provides a steady supply of this potent antioxidant that can halt the propagation of lipid peroxidation and, consequently, cell death [12, 13]. Recent reports have further expanded on the substrate specificity of FSP1 by showing that it can reduce vitamin K to hydroquinone (VKH2) [17, 18]. The significance of FSP1 in regulating ferroptosis and the possibility of targeting FSP1 as a potential sensitizing strategy have recently attracted increasing interest, as exemplified by a number of studies demonstrating FSP1-mediated ferroptosis-resistance to therapy in models of *KEAP1* and *KRAS* mutant lung cancers [19, 20]. Therefore, pharmacological targeting of FSP1 could be exploited to improve ferroptosis-based strategies and radiotherapy.

The first efforts to pharmacologically target FSP1 have been reported in an initial study, where 30.000 drug-like compounds were screened for their capacity to induce cell death in cells where survival is exclusively dependent on FSP1. This study identified iFSP1 as a potent inhibitor of FSP1 [12]. Until now, however, their mode of action has remained largely unexplored. Motivated by this, we present in this study the molecular determinants of the interaction of FSP1 with iFSP1 together with its substrates. These data provide the necessary tools for rationally developing second-generation inhibitors and further provide a cautionary note to its use in animal models, specifically murine models.

## Results

### FSP1-chimeric constructs identify iFSP1-interacting residues

**Fig. 1:**
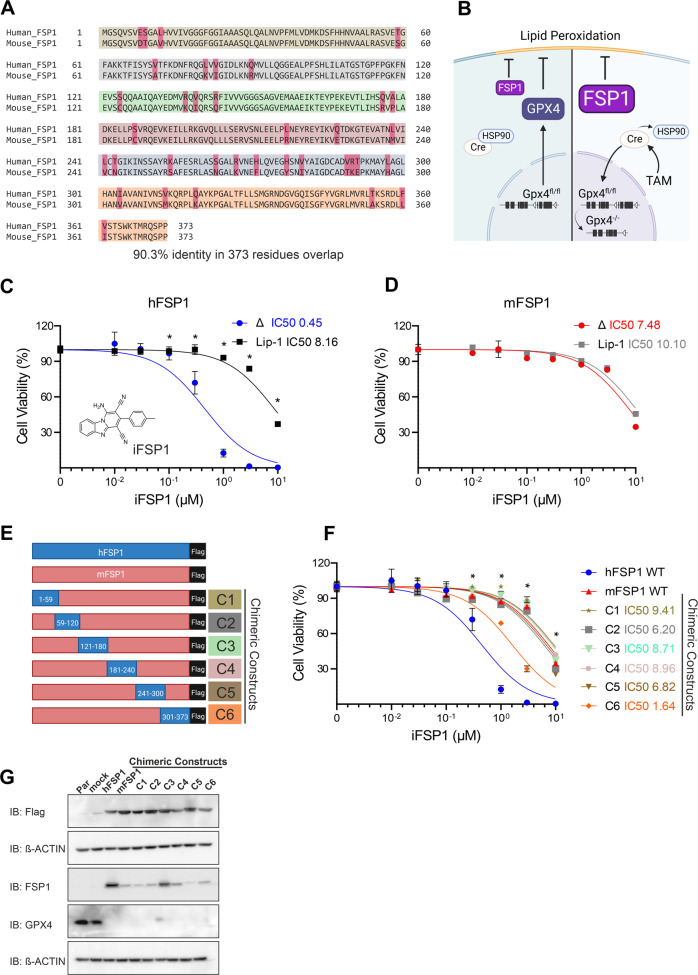
Generation of FSP1 chimeric constructs. **A** Protein sequence pairwise alignment between *Homo sapiens* (Human) and the *Mus musculus* (Mouse) FSP1, the regions used to generate each chimeric construct are highlighted in the different colors. **B** Scheme of GPX4 and FSP1 acting to suppress lipid peroxidation in Tam-inducible *Gpx*4-knockout system (Pfa1 cells). Gpx4, the major suppressor of ferroptosis, with the addition of Tamoxifen (Tam), MerCreMer (Cre) is liberated from the cytosolic HSP90 complex and translocates to the nucleus, where Cre-mediated deletion of the last three loxP-flanked (fl; white triangles) exons (black bars) occurs, leading to inactivation of *Gpx4*. The overexpression of FSP1 inhibits the lipid peroxidation and consequently, cell death. **C** iFSP1 structure. Dose-response toxicity of iFSP1 in Pfa1 *Gpx4*^−/−^ cells overexpressing human FSP1 (hFSP1) and (**D**) murine FSP1 (mFSP1), respectively. **E** Schematic of the different flag-tagged isoforms used to explore the iFSP1 specific response. **F** Dose-response toxicity to iFSP1 in Pfa1 *Gpx4*^−/−^ overexpressing hFSP1, mFSP1 and chimeric variants. **G** Immunoblot analysis of flag-tag, GPX4 and FSP1 in Pfa1 *Gpx4*^WT^ and *Gpx4*^−/−^ overexpressing empty vector (mock), hFSP1, mFSP1 and chimeric proteins. Cell viability was monitored using Alamar blue. Data are presented as mean ± s.d. of *n* = 3 wells of a 96-well plate from one representative of three independent experiments; *p < 0.05; two-way analysis of variance (ANOVA).

The importance of FSP1 for ferroptosis inhibition has been well described, encouraging the characterization and further identification of new inhibitors [12, 13, 19]. Here, we exploit the loss in iFSP1 efficacy on the murine protein as a starting point to identify critically contributing residues. Pairwise sequence alignment between the *Homo sapiens* (Human) and the *Mus musculus* (Mouse) FSP1 (hFSP1 and mFSP1, respectively) allowed us to identify species-specific regions and regions that are identical (90.3% sequence identity) (Fig. 1A).

Using a GPX4 conditional deficient cell line (Pfa1 *Gpx4*^−/−^), we could demonstrate that expression of both the murine and human isoform are functional and able to support viability in the absence of *Gpx4* (Fig. 1B). Nevertheless, iFSP1 could only induce ferroptosis in the cell line expressing the human isoform (Fig. 1C, D) further demonstrating its species-specific effect. The specificity of ferroptosis was validated by rescuing the cells with the ferroptosis inhibitor liproxstatin-1 (Lip-1) (Fig. 1C). To provide additional insights into this feature we designed six chimeric constructs where distinct segments of the murine protein were replaced by the corresponding human counterparts (Fig. 1E). Briefly, chimera (C1) consists of amino acid residue 1 to 59 of the human isoform; the second chimera (C2) replaced residues 59 to 120; the third (C3) residue 121 to 180; the fourth (C4) residues 181 to 240; the fifth (C5) residue 241 to 300 and the last chimera (C6) residues 301 till 373 (Fig. 1A, E). A flag-tag was used in all constructs to ensure reliable detection since the antibody for FSP1 presented differential reactivity to murine and human epitopes on FSP1 (Fig. 1E, G).

All chimeric constructs were overexpressed in the inducible *Gpx4* knockout (KO) cell line (Pfa1) and treated with 4-hydroxytamoxifen (TAM) to generate the respective *Gpx4*^−/−^ cell line expressing the corresponding chimeric FSP1 (Fig. 1B). Next, the activity of iFSP1 to induce ferroptosis was assessed in all cell lines, and while most chimera expressing cells behaved similarly to the iFSP1-insensitive murine isoform, the cell line expressing C6 underwent ferroptosis upon iFSP1 treatment (Fig. 1F). Given that all constructs exhibited similar expression levels, the observed effect for C6 is unlikely related to differences in protein abundance but points to an increased interaction between the inhibitor and this chimeric construct (Fig. 1G).

### F360 is essential for FSP1 inhibition by iFSP1

**Fig. 2:**
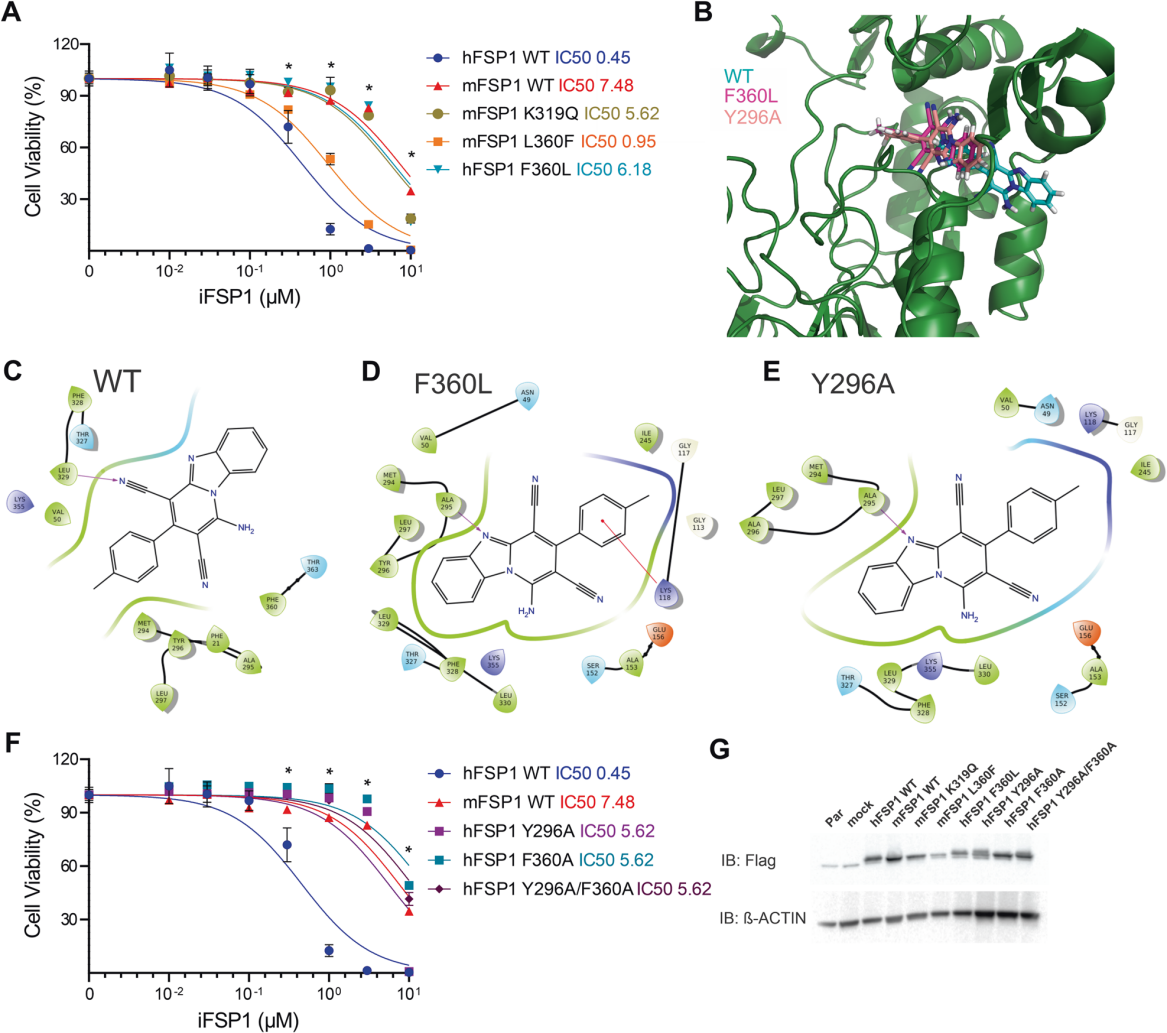
F360 of FSP1 is essential for iFSP1 target engagement. **A** Dose-response toxicity to iFSP1 in Pfa1 *Gpx4*^−/−^ overexpressing hFSP1, mFSP1 and single point mutants (mFSP1^K319Q^, mFSP1^L360F^ and hFSP1^F360L^). **B** Binding modes for iFSP1 obtained from docking the ligand to hFSP1 WT or to different mutants. The backbone of hFSP1 WT is represented in green. Cyan: iFSP1 bound to hFSP1 WT, dark pink: iFSP1 bound to mutants F360L or F360I, light pink: iFSP1 bound to mutants Y296A or F360I/Y296A. Only one FSP1 structure (hFSP1 WT) is represented. Interaction diagrams between iFSP1 and hFSP1 WT (**C**), mutant F360L (**D**) and mutant Y296A (**E**). Purple arrows indicate hydrogen bonds. **F** Dose-response toxicity to iFSP1 in Pfa1 overexpressing hFSP1, mFSP1 and the mutants hFSP1^Y296A^, hFSP1^F360A^ and hFSP1^Y296A/F360A^. **G** Immunoblot analysis of flag-tag, GPX4 and FSP1 in Pfa1 *Gpx4*^−/−^ overexpressing hFSP1, mFSP1 and mutants. Cell viability data was monitored using Alamar blue. Data are presented as mean ± s.d. of *n* = 3 wells of a 96-well plate from one representative of three independent experiments; *p < 0.05; two-way analysis of variance (ANOVA).

Having delineated the region that could be responsible for the interaction between iFSP1 and its target (301–373), we further concentrated on the generation of single amino acid mutations of particular residues. We initially selected amino acids within this region which exhibited the most notable differences in their side chains. To this end, we introduced the following single-point mutations K319Q, and L360F in the murine isoform. Similarly, as presented in the previous section, these constructs were expressed in Pfa1 cells and treated with TAM. Subsequently, we treated the GPX4 deficient cells expressing the FSP1 mutants with iFSP1. Gratifyingly, the L360F mutation restored the iFSP1-mediated inhibition of mFSP1, indicating a critical role of this residue. Additional proof was provided by generating the corresponding F360L mutation in the human isoform. Accordingly, this single modification completely abolished the capacity of iFSP1 to induce ferroptosis in these cells (Fig. 2A).

### Molecular modeling of iFSP1 binding identifies critical species-specific aromatic interactions

To rationalize the binding of iFSP1 to the different hFSP1 mutants, we conducted molecular docking studies. Our results suggest that when iFSP1 is bound to hFSP1 WT, the ligand engages in a pi-pi stacking interaction with F360, Y296 and F21, and a hydrogen bond with L329. During the binding of iFSP1 to the mutant F360L, as F360 is no longer available, the stacking interaction is lost, reducing the binding energy of the ligand. To validate the pi-pi stacking between these residues and iFSP1, we explored the consequences in molecular docking using mutants of hFSP1, where F21, F360 or/and Y296 were mutated to Alanine. The docking score obtained for iFSP1 bound to hFSP1 WT was lower than that obtained for iFSP1 bound to all mutants F21A, Y296A, F360L/A and the double or triple mutants (Supplementary Table 1). Docking scores attempt to correlate with binding free energy, suggesting a higher affinity of iFSP1 for hFSP1 WT. Moreover, the proposed binding models obtained for iFSP1 were remarkably distinct between the WT and the FSP1 mutants (Fig. 2B), suggesting that the ligand is unable to engage in a stable interaction with the protein in the mutant form (Fig. 2C–E). Inspired by this, we generated single and double alanine mutations for these positions to experimentally validate importance of the interactions involving F360, F296 and iFSP1. Using these constructs, we overexpressed the mutants hFSP1^Y296A^, hFSP1^F360A^ and hFSP1^Y296A/F360A^ in Pfa1 *Gpx4*^−/−^ and treated the cells with iFSP1, which confirmed the computational predictions by showing that absence of these stacking interactions abolishes the inhibitor activity (Fig. 2F, G).

**Fig. 3:**
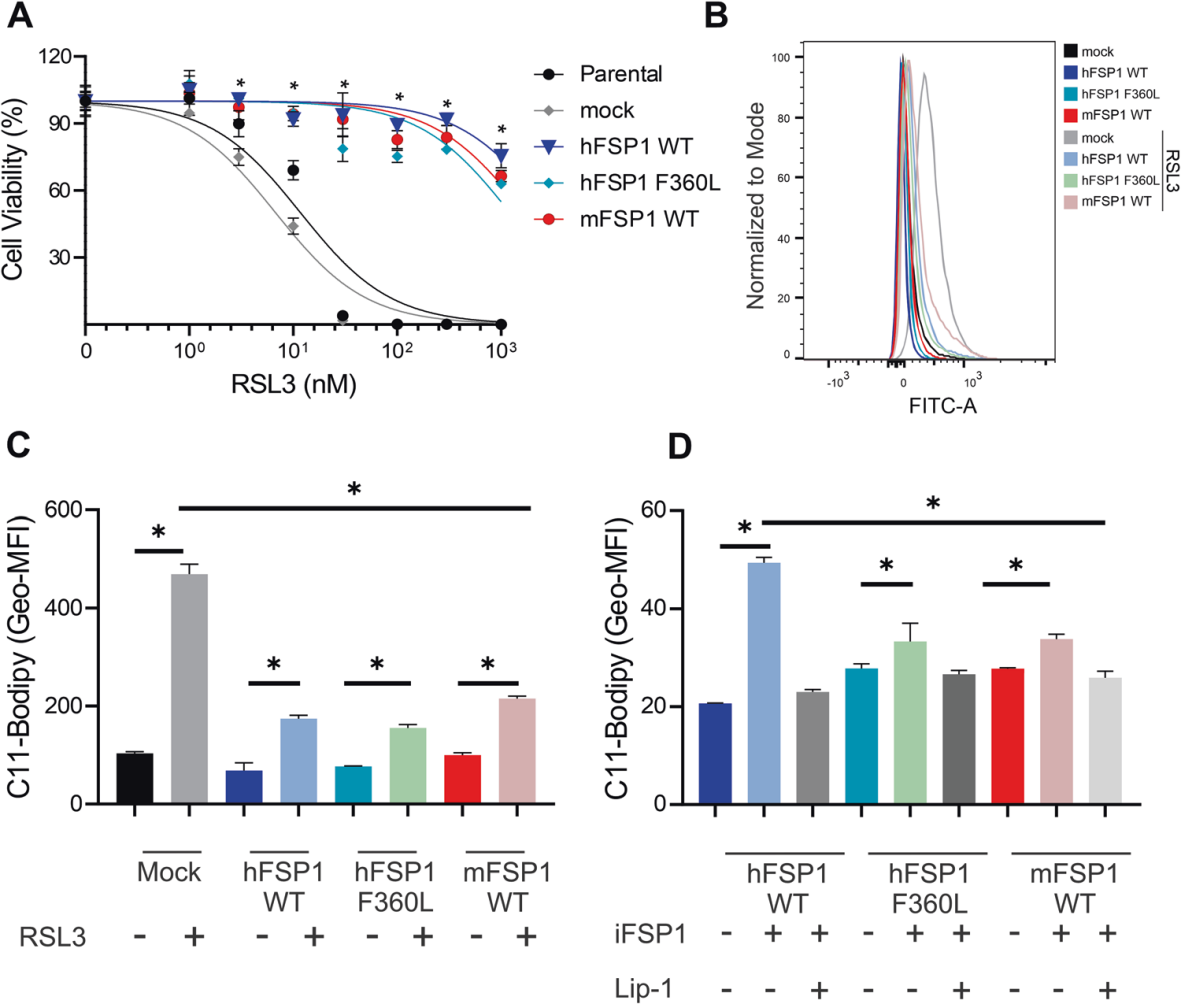
F360L mutation does not interfere with FSP1 activity. **A** Dose-response toxicity to iFSP1 in Pfa1 *Gpx4*^WT^ overexpressing the hFSP1, mFSP1 and hFSP1^F360L^ mutant. Cell viability data was monitored using Alamar blue. Data are presented as mean ± s.d. of *n* = 3 wells of a 96-well plate from one representative of three independent experiments; *p < 0.05; two-way analysis of variance (ANOVA). Flow cytometry analysis of BODIPY 581/591 C11 oxidation induced by RSL3 treatment (300 nM for 3 h) in Pfa1 *Gpx4*^WT^cell line overexpressing hFSP1, mFSP1 and hFSP1^F360L^ mutant. **B** Histogram (**C**) bar graphs of the geometric mean fluorescence intensity (Geo-MFI). **D** Flow cytometry analysis of BODIPY 581/591 C11 oxidation induced by iFSP1 treatment (3 µM for 3 h) with or without liproxstatin 1 (Lip-1) in Pfa1 *Gpx4*^−/−^ cell line overexpressing hFSP1, mFSP1 and hFSP1^F360L^ mutants. Bar graphs depict the geometric mean fluorescence intensity (Geo-MFI). Data are presented as mean ± s.d. of *n* = 3 wells of one representative of two independent experiments; *p < 0.05; one-way analysis of variance (ANOVA) with post-hoc Tukey.

Further characterization of the hFSP1^F360L^ variant was pursued to exclude unaccounted effects associated to the biology of FSP1. To exclude the potential impact on FSP1 activity, we treated the Pfa1 cells overexpressing the mock, hFSP1^WT^, hFSP1^F360L^ and mFSP1^WT^ with the GPX4 inhibitor (1 S,3 R)-RSL3 (RSL3), where we observed no difference in response to the GPX4 inhibitor between the cells overexpressing the FSP1 variants (Fig. 3A). We also evaluated BODIPY 581/591 C11 oxidation as a proxy of phospholipid peroxidation (pLPO) and found that hFSP1^WT^, hFSP1^F360L^ and mFSP1^WT^ overexpression prevented BODIPY oxidation upon RSL3 to the same extent (Fig. 3B, C). BODIPY-C11 oxidation induced by iFSP1 inhibition in GPX4 deficient cells was also evaluated in cells overexpressing hFSP1^WT^, hFSP1^F360L^ and mFSP1^WT^. Under these conditions, a small increase was observed in all conditions being the highest for hFSP1^WT^. In all conditions, the increase in BODIPY-C11 signal could be rescued by the ferroptosis inhibitor Lip-1, suggesting that they are indeed arising from lipid peroxidation (Fig. 3D). Further proof that the inhibitory effect is specific to the iFSP1/FSP1 interaction was demonstrated using a system where FSP1 disruption is achieved by interfering with its subcellular localization. We previously showed that myristoylation of FSP1 is essential for its anti-ferroptotic activity [12]. As such, we treated the Pfa1 *Gpx4*^−/−^ overexpressing hFSP1^WT^, hFSP1^F360L^ and mFSP1^WT^ with the N’Myristoyltransferase inhibitor IMP-1088. These experiments showed that all mutants undergo ferroptosis similarly and that the F360L is only critical to binding with iFSP1 (Supplementary Fig. 1A).

### FSP1-Tracer determines iFSP1 affinity and ligand binding

**Fig. 4:**
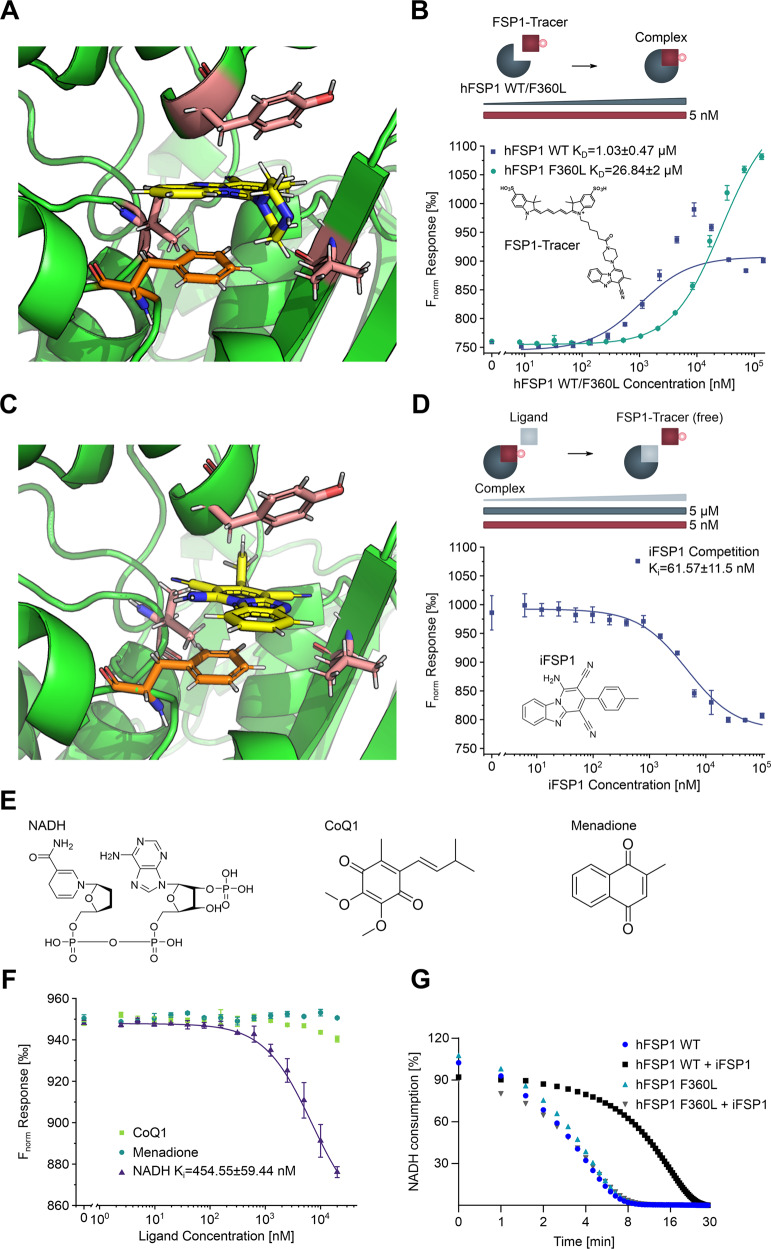
Biophysical binding affinity quantification of iFSP1 to hFSP1 WT/F360L. **A** Molecular docking of iFSP1-Pip into hFSP1 WT. hFSP1: green, iFSP1: yellow, relevant residues for binding: salmon, F360: orange. Note that the piperazine presents an exit vector and protrudes from the binding pocket. **B** Direct binding affinity of FSP1-Tracer to hFSP1 WT and F360L. Increasing concentrations of hFSP1 WT and F360L were titrated against FSP1-Tracer. Note the 26-fold reduction in binding affinity for hFSP1 F360L. Data are presented as mean of *n* = 4 measurements with standard deviation. **C** Molecular docking of iFSP1 into hFSP1 WT. hFSP1: green, iFSP1: yellow, relevant residues for binding: salmon, F360: orange. **D** Competition MST to quantify binding affinity of iFSP1 to hFSP1 WT. Increasing concentrations of iFSP1 were titrated against a pre-formed complex of hFSP1 WT and FSP1-Tracer (5 µM and 5 nM respectively) to obtain a K_i_ value for the binding affinity. Data are presented as mean of *n* = 3 measurements with standard deviation. **E** Structures of three known substrates of FSP1 (NADH, CoQ1, Menadione). **F** Competition MST to quantify binding affinity of NADH, CoQ1 and Menadione to hFSP1 WT. Increasing concentrations of the three compounds were titrated against a pre-formed complex of hFSP1 WT and FSP1-Tracer (5 µM and 5 nM respectively) to obtain a K_i_ value for the binding affinity. Data are presented as mean of *n* = 3 measurements with standard deviation. **G** NADH consumption assay (340 nm) in TBS buffer using recombinant purified hFSP1 WT and F360L mutant with and without iFSP1 (2 µM), using menadione as substrate. Data are presented as mean of *n* = 2 technical replicates of one out of three independent experiments.

Having established a critical role for residue F360 within hFSP1 for iFSP1 binding in vitro and in silico, we sought to determine the interaction strength of the inhibitor with the WT protein and the F360L mutant. To this end, we established a quasi-label-free, microscale thermophoresis (MST)-based displacement assay [21] using a novel fluorescent tracer to circumvent covalent fluorescent labeling of FSP1 and preserve structural integrity. The tracer was designed to bind the same binding pocket as iFSP1 and harbor a fluorescent dye for effective detection in MST assays while still displaying high water solubility. This was achieved using the minimal iFSP1 pharmacophore with a piperazine group (iFSP1-Pip) as an exit vector protruding from the FSP1 binding pocket (Fig. 4A) for conjugation to a SulfoCyanine5 [22]. The resulting fluorescent tracer “FSP1-Tracer” enabled the detection of FSP1 binding and further highlights the precursor as starting point for the design of advanced iFSP1-based effectors.

Next, we used FSP1-Tracer to determine its direct binding affinity towards recombinantly expressed hFSP1 WT and F360L. We observed a 26-fold reduction in binding affinity (1.03 µM versus 26.84 µM) for the F360L variant compared to the WT protein, thus unequivocally revealing the critical importance of F360 for iFSP1 binding (Fig. 4B). These observations are in good agreement with the reported values observed in the cellular experiments. Next, a displacement assay was performed, where a pre-formed complex of hFSP1 WT and FSP1-Tracer was exposed to increasing concentrations of iFSP1. Based on the previously determined direct binding affinity and the employed concentrations of hFSP1 and the fluorescent tracer, a K_i_ value of 61.57 nM was determined (Fig. 4C), closely matching the nM EC_50_ values of iFSP1 that were observed in cells (Fig. 1C). To further explore the mechanism of action of iFSP1, we conducted competition assays with three known interactors of FSP1, namely NADH and water-soluble analogs of CoQ10 and vitamin K, CoQ1 and menadione respectively (Fig. 4E). We envisioned that competition of one of these substrates with FSP1-Tracer in complex with hFSP1 WT would indicate that they occupy the same binding pocket within the protein. Curiosly, in the case of NADH but not CoQ1 or menadione, a K_i_ value of 454.55 nM was determined, suggesting that iFSP1 exerts its function by competitively inhibiting NADH binding, having a 7.4-fold higher binding affinity to hFSP1.

Finally, using recombinantly expressed hFSP1 WT and F360L, we performed a direct enzymatic assay using menadione as a substrate and compared their ability to reduce NADH. Notably, no difference could be observed between the activities of both proteins. As expected, just the activity of the WT protein was inhibited by iFSP1 (Fig. 4G). Similar results were observed when using ubiquinone (CoQ1) or resazurin (Supplementary Fig. 1B, C) as substrates for the enzyme.

Taken together, we provide unequivocal validation of the involvement of F360 in iFSP1 binding, offer a biophysical quantification of the interaction between FSP1 and iFSP1 and provide novel insights into the mechanism of FSP1 inhibition by this class of compounds.

## Discussion

Mechanisms interfering with the cell’s capacity to detoxify peroxidized phospholipids have gained increasing attention as a way to induce ferroptosis in therapeutically challenging cancer entities. Specifically, efforts have centered on interfering with glutathione-dependent pathways, including cysteine uptake, reduced glutathione (GSH) biosynthesis and directly inhibiting the GSH-utilizing enzyme GPX4. Nevertheless, it has become increasingly accepted that this might not be sufficient, and backup mechanisms regulating and inhibiting lipid peroxidation do exist and need to be suppressed for efficient ferroptosis induction [12, 13, 15]. One key mechanism requires the flavoprotein FSP1, a dedicated oxidoreductase involved in the regeneration of lipophilic antioxidants. Our previous work has shown that the dual targeting of GPX4 and FSP1 is a potent strategy for the induction of ferroptosis and will likely be required for triggering this form of cell death in preclinical models. These findings motivated the studies that led to the identification of a potent small molecule inhibitor of FSP1, iFSP1 [12], yet despite this initial identification, its mechanism of action has remained largely unexplored.

Our present study shows that iFSP1 only exerts its function on hFSP1 but not mFSP1. Given the increased use of iFSP1 as an FSP1 inhibitor, and its indiscriminate use in murine models, our results provide a cautionary note for the interpretation of the results obtained with this compounds and will be important to establish their proper use. Motivated by this observation, we went forward using a series of chimeras and mutational studies that allowed us to pinpoint critical residues within hFSP1 that mediate binding to iFSP1. Using a GPX4 conditional KO cell model, we could identify the residue F360 within human FSP1 to be critically involved in the engagement of iFSP1 with its target. Furthermore, our results demonstrate that disruption of any amino acid involved in the aromatic pi-pi stacking interactions occurring between hFSP1 and iFSP1 severely impairs iFSP1 interaction and, consequently, FSP1 inhibition. The biophysical quantification of the in vitro and in silico characterized interaction was initially hampered by the poor water solubility of iFSP1, which rendered it impossible to determine the dissociation constant using methodologies that require comparably high ligand concentrations in solution, such as isothermal titration calorimetry (ITC). To circumvent this, a quasi-label-free approach was established with FSP1-Tracer, which was designed based on the in silico dockings (Fig. 4A). This allowed for direct affinity quantification and competition assays with iFSP1 and the three known ligands of hFSP1, which provided insights into the binding mechanism. This novel tool could be of fundamental importance for the design of inhibitors in the future. Vice versa, FSP1-Tracer also provides an attractive architecture with a handle for the design of future iFSP1-based degraders that will rely on a viable exit vector to conjugate possible conventional or novel E3-ligase recruiters.

Curiously, the results obtained with FSP1-Tracer suggest that NADH is able to compete with iFSP1 for binding in the active site of FSP1. This is unexpected as studies describing the cofactor binding pockets of NDH2, a highly homologous type II NADH:quinone oxidoreductase [23], suggested a higher degree of overlap in iFSP1 binding with the proposed quinone binding pocket. Yet, this could suggest a different binding mechanism of NADH within FSP1 and further studies will be required to clarify this.

In sum, our study identified the binding site of iFSP1 within its target, thus providing insights into its exact binding mechanism, which will enable the development of rationally designed second-generation FSP1 inhibitors and the development of proteolysis targeting chimeras that could ultimately be critical to advance our understanding and the use of ferroptosis based strategies in preclinical settings.

## Materials and methods

Cell Lines. 4-hydroxytamoxifen (TAM)-inducible *Gpx4*^−/−^ murine immortalized fibroblasts (Pfa1) were reported previously [24]. These cells harbor two loxP-flanked *Gpx4* alleles and stably express TAM inducible Cre recombinase allowing the genetic deletion of *Gpx4* at will. For all experiments using *Gpx4*^−/−^ the well were treated with 4-hydroxitamoxifen (TAM) 1 µM. All cells were cultured with were cultured in DMEM-high glucose (4.5 g glucose/L) with 10% fetal bovine serum (FBS), Glutamax, and 1% penicillin/streptomycin (Gibco, Thermo Fischer) at 37 °C with 5% CO_2_ and verified to be negative for mycoplasma.

Cell viability assays. Alamar Blue method: Cells were seeded on 96-well plates (2000 cells per well) and treated with the compounds RSL3, iFSP1 and (liproxstatin-1) after plating. Cell viability was assessed 48 h (unless stated otherwise) after treatment using Alamar Blue as an indicator of viable cells. Alamar blue solution was made by dissolving of 1 g resazurin sodium salt (Thermo Fischer) in 200 mL sterile PBS and sterile filtrated through a 0.22 µm filter. Stock solutions were stored at 4 °C. The working solution was made freshly by adding 200 µL of the stock solution to 25 mL growth media. After 2–4 h incubation time, viability was estimated by measuring the fluorescence using a 540/35 excitation filter and a 590/20 emission on a Spark® microplate reader (Tecan, Zürich, Switzerland).

### Construction of plasmids

Codon-optimized *Mus musculus* (mouse) FSP1 (NP_001034283.1) and *Homo sapiens* (Human) FSP1 (NP_001185625.1) were synthesized by IDT as gBlocks^TM^ and subcloned into p442-IRES-blast vectors. The chimera constructs were generated based on the pairwise alignment between the gene of *Homo sapiens* (Human) and the *Mus musculus* (Mouse), all the chimeric constructs contain the murine isoform with one region exchanged to the human isoform. The chimera (C1) consist of residue 1 to 59 corresponding to human isoform of the human gene; the second chimera (C2) replaced residues 59 to 120; the third (C3) residue 121 to 180; the fourth (C4) residues 181 to 240; the fifth (C5) residue 241 to 300 and the last chimera (C6) residues 301 till 373. The chimeric constructs were synthesized by IDT and subcloned into p442-IRES-blast vectors. All constructs contain a flag-tag.

### Preparation of lentiviral particles for overexpression of genes

HEK 293 T cells were used to produce replication-incompetent lentiviral particles pseudotyped with a third generation lentiviral packaging system consisting of three plasmids (ecotropic particles - pHCMV-EcoEnv, packing - pMDLg/pRRE and pRSV-Rev) and the was co-lipofected into HEK 293 T cells using X-tremeGENE HP DNA Transfection Reagent (Roche). Viral particle containing cell culture supernatants were harvested 48 h after transfection and used to transduce the Pfa1 cell line incubating cell with HEK293T supernatants filtered through a 0.44 µM membrane.

### Immunoblotting

Cells were lysed in RIPA lysis buffer (0.5% Triton X-100, 0.5% sodium deoxocholate salt, 150 mM NaCl, 20 mM TRIS, 10 mM EDTA, and 30 mM Na-pyrophosphate [pH 7.5]), containing a protease inhibitor cocktail (Roche, Mannheim, Germany). Protein concentration was determined by Pierce™ BCA Protein Assay Kit (ThermoFischer). Western blotting analysis was performed with a GPX4 monoclonal antibody (1:1000; no. ab125066, Abcam), Flag-Tag monoclonal antibody (1:2500 no. F3165 - Sigma-Aldrich), β-ACTIN (1:5000; no. A5441, Sigma) or human FSP1 (1:5, rat IgG2a, clone AIFM2 6D8, developed in-house). Chemiluminescent images were acquired on a chemiluminescent detection system (Azure 300, Biozym, Germany). Full and uncropped western blots are presented in Supplementary File.

### Modeling of FSP1

An initial model of the human FSP1 was obtained from the AlphaFold Protein Structure Database [25, 26]. In the final model of FSP1, residues 1 to 10 were removed due to a low model confidence (per-residue confidence score, pLDDT, lower than 58). Structure validation was performed using MolProbity, implemented in the SWISS-MODEL web server [27]. The modeled structure achieved a MolProbity score of 0.96. 97.78% of the residues were positioned in favored regions of the Ramachandran plot, and 0% were positioned in outlier regions (Supplementary Fig. 2). Prior docking, the protonation states of the residues at pH 7.4 were determined using PROPKA 3 [28, 29] as implemented in PDB2PQR 2.1.1 [30, 31]. The mutants F21A, Y296A, F360L/I, F21A/Y296A, F21A/F360A, Y296A/F360A/L and F21A/Y296A/F360A of hFSP1 were built using PyMol (Version 1.8.4.0).

### Docking of iFSP1 and iFSP1-Pip to FSP1

Docking of iFSP1 to FSP1 (WT or mutants) was performed with AutoDock Vina [32]. An exhaustiveness level of 8 and a cubic grid with a spacing of 0.375 Å, 80 points along each edge and center around the alpha carbon of residue 360 were used for docking. Bond rotations were allowed in the ligand, while the protein structures were kept rigid. 20 ligand poses were generated by docking, and the top scoring pose was selected.

### Protein expression and purification

FSP1 (human isoform 1, UniProt Q9BRQ8-1) WT (hFSP1 WT) and F360L (hFSP1 F360L) were expressed in *E.coli* BL21 as HisSUMO fusion protein. Cells were grown in lysogeny broth medium at 18 °C and induced with 0.5 mM isopropyl-β-thiogalactoside at a cell density A_600_ of 0.6. Cells were collected after overnight expression by centrifugation, resuspended in lysis buffer (300 nM NaCl, 50 mM Tris-HCl pH 8, 1 mM TCEP, 20 mM imidazole, protease inhibitor, DNase) and lysed by sonication. Cell debris was removed by centrifugation (30,000 × *g*) at 4 °C for 30 min, after which an IMAC purification was performed. Briefly, a HisTrap crude FF (5 mL) column was equilibrated to lysis buffer, lysates were loaded via the sample pump and the column was then washed with 50 mL lysis buffer. During the elution with a gradient to 100% IMAC elution buffer (300 mM NaCl, 50 mM Tris-HCl pH 8, 1 mM TCEP, 400 mM imidazole) over 100 mL, 5 mL fractions were collected. The pooled fractions were dialysed overnight in the presence of SenP2 SUMO protease at a ratio of 40:1 FSP1:SenP2. Afterwards, a reverse IMAC purification was performed on a HisTrap crude FF (5 mL) column. IMAC eluates were analysed by SDS PAGE and concentrated to 15 mL (hFSP1 WT) and 5 mL (hFSP1 F360L), respectively. Afterwards, the samples were applied to a 26/600 Superdex 200 (hFSP1 WT) or a 16/600 Superdex 200 (hFSP1 F360L) size exclusion column equilibrated with 50 mM Tris-HCl pH 8, 300 mM NaCl, 0.5 mM TCEP (Supplementary Fig. 3). Pure fractions were pooled (C12—D11 for hFSP1 WT and C7—D4 for hFSP1 F360L), analysed by SDS-PAGE (Supplementary Fig. 4) and concentrated to 5.8 mL at 285.9 µM (hFSP1 WT) and 1.2 mL at 269.5 µM (FSP1 F360L), flash frozen in liquid N_2_ and stored at −80 °C.

### Assessment of lipid peroxidation using C11-BODIPY (581/591)

In total, 150,000 cells per well were seeded on 6-well dishes one day prior to the experiment. On the next day, cells were treated with 300 nM of RSL3 or 3 µM of iFSP1 to induce ferroptosis. Cells were incubated with C11-BODIPY (581/591) (1 μM) for 30 min at 37 °C before they were harvested by trypsinisation. Subsequently, cells were resuspended in 500 μL of fresh PBS (DPBS, Gibco) and analyzed using a 488-nm laser excitation laser and fluorescence recorded on a (FACS Canto II, BD Biosciences). Data was collected from the FL1 detector (C11-BODIPY, Invitrogen) with a 502LP and 530/30 BP filter. At least 20,000 events were analyzed per sample. Data was analyzed using FlowJo Software version 10.8.1.

### FSP1-tracer synthesis

A minimal pharmacophore with a piperazine handle (iFSP1-Pip) was obtained from ChemBridge Corporation (San Diego, CA), SCy5 NHS-ester was obtained from Lumiprobe GmbH (Hannover, DE) and both were used without further purification. Amide coupling of iFSP1-Pip to SCy5 NHS-ester was performed in dimethyl sulfoxide (DMSO) with 10 equivalents of N,N-Diisopropylethylamine (DIEA) for 16 h at 25 °C under agitation. The crude reaction mixture was purified by high performance liquid chromatography (HPLC) to >99% purity, as determined by liquid chromatography-mass spectrometry (LC-MS) (Supplementary Fig. 5).

### Microscale thermophoresis measurements

An MST buffer consisting of 1× phosphate-buffered saline (PBS; 137 mM NaCl, 2.7 mM KCl, 10 mM Na2HPO4, 1.8 mM KH2PO4, pH 7.4) with 2 mM reduced L-Glutathione (GSH, Sigma Aldrich, product number G4251) and 0.05% (v/v) Tween 20 (ITW Reagents, molecular biology grade, product number A4974) was used for all MST measurements [33]. Prior to binding assays, hFSP1 WT and F360L proteins were buffer exchanged to MST buffer using 7 K MWCO ZebaTM Spin desalting columns (ThermoScientific) according to the manufacturer’s instructions. For measurements of direct binding affinity, 15-point, 2-fold dilution series of hFSP1 WT/F360L were prepared (10 µL per dilution) with an additional negative control containing no protein, after which 10 µL of a 10 nM solution of FSP1-Tracer was added. For competition measurements, a 15-point, 2-fold dilution series of iFSP1 and a 14-point, 2-fold dilution series for NADH, CoQ1 or Menadione in MST buffer was prepared (10 µL per dilution) with an additional negative control containing no ligand. Afterwards, 10 µL of a solution of 10 nM FSP1-Tracer and 10 µM hFSP1 WT that was pre-incubated on ice for 15 min was added to each sample. Samples were then incubated for 15 min at room temperature and then transfered to standard MST capillaries (Nanotemper Technologies GmbH, Munich, DE). Measurements were carried out on a Monolith NT.115 (Nanotemper Technologies GmbH, Munich, DE) with Pico Red detection. Settings were 1% excitation power, medium MST power and signal evaluation at 10 s. Data were plotted in OriginPro 2021 9.8.0.200 (OriginLab, Northampton, MA). K_D_ values were determined by applying a Hill-fit to a plot of F_norm_ vs. hFSP1 WT/F360L concentration. For competition assays, the EC_50_ value was first determined by applying a Hill-fit to a plot of F_norm_ vs. ligand concentration, after which formula (1) and (2) were used to calculate the respective K_i_ value.

1 $$K_i = \frac{{K_d}}{{2 - \gamma }} \cdot \left( {\frac{{EC_{50}}}{{\frac{{\left[ T \right]_t}}{\gamma } - \frac{{K_d}}{{2 - \gamma }} - \frac{{\left[ C \right]_t}}{2}}} - \gamma } \right)$$

with

2 $$\gamma = \frac{{\left[ T \right]_t + \left[ C \right]_t + K_d - \sqrt {(\left[ T \right]_t + \left[ C \right]_t + K_d)^2 - 4\left[ T \right]_t\left[ C \right]_t} }}{{2\left[ C \right]_t}}$$

and

- [*T*]_*t*_ The total final concentration of the unlabeled target (hFSP1 WT/F360L) in the assay
- [*C*]_*t*_ The total final concentration of fluorescent tracer (FSP1-Tracer) in the assay that forms a complex with the target protein and is replaced by unlabeled iFSP1, NADH, Menadione or CoQ1
- *K*_*D*_ The K_D_ between the fluorescent tracer and the target protein from a direct binding affinity measurement
- *EC*_50_ The EC_50_ obtained from titrating an unlabeled ligand against the preformed complex of target protein and tracer

### FSP1 enzyme activity and inhibition assay

Enzyme reactions were performed in TBS buffer (50 mM TRIS pH 8, 250 mM NaCl) on a 96-well plate in a 100 µL final volume. The reactions contained 700 nM of human recombinant FSP1 protein WT or F360L, 500 μM NADH (freshly prepared in TBS buffer) and 200 μM of the following substrates: CoQ1 (Ubiquinone1, Sigma), Menadione (Vitamin K3, Sigma) or (Resazurin, Sigma). Absorbance (340 nm) was recorded every 30 s on a Spark® microplate reader (Tecan, Zürich, Switzerland) in order to determine NADH consumption. Reactions without NADH/ without enzyme were used to normalize the results.

### Data presentation and statistical analyses

All data are expressed as the mean of triplicate measures ± standard deviation (SD) unless otherwise stated. GraphPad Prism 9.5.0 was used to perform statistical analysis if not stated otherwise. The graph shows representative of a single experiment performed two or three for reproducibility, shown in the figure legends. Statistical analyses of samples were performed using one-way or two-way ANOVA with Tukey’s post-hoc test. In all cases, significance is indicated as follows: *p < 0.05.

## Supplementary information

Suppl_Info

Original Data File

Reproducibility checklist

## Data Availability

Materials and the experimental data sets generated here are available from the corresponding author upon reasonable request. No applicable resources were generated during the current study.

## References

[1] Dixon SJ, Patel DN, Welsch M, Skouta R, Lee ED, Hayano M (2014). Pharmacological inhibition of cystine-glutamate exchange induces endoplasmic reticulum stress and ferroptosis. Elife..

[2] Stockwell BR (2022). Ferroptosis turns 10: emerging mechanisms, physiological functions, and therapeutic applications. Cell..

[3] Nishida Xavier da Silva T, Friedmann Angeli JP, Ingold I (2022). GPX4: old lessons, new features. Biochem Soc Trans.

[4] Hangauer MJ, Viswanathan VS, Ryan MJ, Bole D, Eaton JK, Matov A (2017). Drug-tolerant persister cancer cells are vulnerable to GPX4 inhibition. Nature..

[5] Viswanathan VS, Ryan MJ, Dhruv HD, Gill S, Eichhoff OM, Seashore-Ludlow B (2017). Dependency of a therapy-resistant state of cancer cells on a lipid peroxidase pathway. Nature..

[6] Tsoi J, Robert L, Paraiso K, Galvan C, Sheu KM, Lay J (2018). Multi-stage differentiation defines melanoma subtypes with differential vulnerability to drug-induced iron-dependent oxidative stress. Cancer Cell.

[7] Mai TT, Hamai A, Hienzsch A, Caneque T, Muller S, Wicinski J (2017). Salinomycin kills cancer stem cells by sequestering iron in lysosomes. Nat Chem.

[8] Weigand I, Schreiner J, Rohrig F, Sun N, Landwehr LS, Urlaub H (2020). Active steroid hormone synthesis renders adrenocortical cells highly susceptible to type II ferroptosis induction. Cell Death Dis.

[9] Friedmann Angeli JP, Schneider M, Proneth B, Tyurina YY, Tyurin VA, Hammond VJ (2014). Inactivation of the ferroptosis regulator Gpx4 triggers acute renal failure in mice. Nat Cell Biol.

[10] Yang WS, SriRamaratnam R, Welsch ME, Shimada K, Skouta R, Viswanathan VS (2014). Regulation of ferroptotic cancer cell death by GPX4. Cell..

[11] Friedmann Angeli JP, Conrad M (2018). Selenium and GPX4, a vital symbiosis. Free Radic Biol Med.

[12] Doll S, Freitas FP, Shah R, Aldrovandi M, da Silva MC, Ingold I (2019). FSP1 is a glutathione-independent ferroptosis suppressor. Nature..

[13] Bersuker K, Hendricks JM, Li Z, Magtanong L, Ford B, Tang PH (2019). The CoQ oxidoreductase FSP1 acts parallel to GPX4 to inhibit ferroptosis. Nature.

[14] Kraft VAN, Bezjian CT, Pfeiffer S, Ringelstetter L, Muller C, Zandkarimi F (2020). GTP cyclohydrolase 1/tetrahydrobiopterin counteract ferroptosis through lipid remodeling. ACS Cent Sci.

[15] Soula M, Weber RA, Zilka O, Alwaseem H, La K, Yen F (2020). Metabolic determinants of cancer cell sensitivity to canonical ferroptosis inducers. Nat Chem Biol.

[16] Barayeu U, Schilling D, Eid M, Xavier da Silva TN, Schlicker L, Mitreska N (2023). Hydropersulfides inhibit lipid peroxidation and ferroptosis by scavenging radicals. Nat Chem Biol.

[17] Mishima E, Ito J, Wu Z, Nakamura T, Wahida A, Doll S (2022). A non-canonical vitamin K cycle is a potent ferroptosis suppressor. Nature..

[18] Jin DY, Chen X, Liu Y, Williams CM, Pedersen LC, Stafford DW (2023). A genome-wide CRISPR-Cas9 knockout screen identifies FSP1 as the warfarin-resistant vitamin K reductase. Nat Commun.

[19] Koppula P, Lei G, Zhang Y, Yan Y, Mao C, Kondiparthi L (2022). A targetable CoQ-FSP1 axis drives ferroptosis- and radiation-resistance in KEAP1 inactive lung cancers. Nat Commun.

[20] Muller F, Lim JKM, Bebber CM, Seidel E, Tishina S, Dahlhaus A (2023). Elevated FSP1 protects KRAS-mutated cells from ferroptosis during tumor initiation. Cell Death Differ.

[21] Schulte C, et al. High-throughput determination of protein affinities using unmodified peptide libraries in nanomolar scale. Iscience. 2021;24:101898. 10.1016/j.isci.2020.101898 PMC7753147 33364586

[22] Schulte C, et al. Multivalent binding kinetics resolved by fluorescence proximity sensing. Communications biology. 2022;5:1070. 10.1038/s42003-022-03997-3 PMC9546861 36207490

[23] Marreiros BC, Sena FV, Sousa FM, Oliveira AS, Soares CM, Batista AP (2017). Structural and functional insights into the catalytic mechanism of the Type II NADH:quinone oxidoreductase family. Sci Rep..

[24] Seiler A, Schneider M, Forster H, Roth S, Wirth EK, Culmsee C (2008). Glutathione peroxidase 4 senses and translates oxidative stress into 12/15-lipoxygenase dependent- and AIF-mediated cell death. Cell Metab.

[25] Jumper J, Evans R, Pritzel A, Green T, Figurnov M, Ronneberger O (2021). Highly accurate protein structure prediction with AlphaFold. Nature..

[26] Varadi M, Anyango S, Deshpande M, Nair S, Natassia C, Yordanova G (2022). AlphaFold Protein Structure Database: massively expanding the structural coverage of protein-sequence space with high-accuracy models. Nucleic Acids Res.

[27] Waterhouse A, Bertoni M, Bienert S, Studer G, Tauriello G, Gumienny R (2018). SWISS-MODEL: homology modelling of protein structures and complexes. Nucleic Acids Res.

[28] Li H, Robertson AD, Jensen JH (2005). Very fast empirical prediction and rationalization of protein pKa values. Proteins..

[29] Olsson MH, Sondergaard CR, Rostkowski M, Jensen JH (2011). PROPKA3: consistent treatment of internal and surface residues in empirical pKa predictions. J Chem Theory Comput.

[30] Dolinsky TJ, Nielsen JE, McCammon JA, Baker NA (2004). PDB2PQR: an automated pipeline for the setup of Poisson-Boltzmann electrostatics calculations. Nucleic Acids Res.

[31] Unni S, Huang Y, Hanson RM, Tobias M, Krishnan S, Li WW (2011). Web servers and services for electrostatics calculations with APBS and PDB2PQR. J Comput Chem.

[32] Trott O, Olson AJ (2010). AutoDock Vina: improving the speed and accuracy of docking with a new scoring function, efficient optimization, and multithreading. J Comput Chem.

[33] Schulte C, et al. Low-cost synthesis of peptide libraries and their use for binding studies via temperature-related intensity change. STAR protocols. 2021;2:100605. 10.1016/j.xpro.2021.100605 PMC8219886 34189471

