# Supplementary material for: Molecular characterization of AIFM2/FSP1 inhibition by iFSP1-like molecules
Source: Cell Death Dis. 2023 Apr 21;14(4):281. doi: 10.1038/s41419-023-05787-z (PMC10119282; doi:10.1038/s41419-023-05787-z)
Supplement: Supplementary file 1 — Suppl_Info [file 41419_2023_5787_MOESM1_ESM.docx]

# Supplementary Information

**Molecular characterization of AIFM2/FSP1 inhibition by iFSP1-like molecules**

Thamara Nishida Xavier da Silva^1^; Clemens Schulte^1^; Ariane Nunes Alves^2^, Hans Michael Maric^1^; José Pedro Friedmann Angeli^1^*

*Correspondence to [pedro.angeli@virchow.uni-wuerzburg.de](mailto:pedro.angeli@virchow.uni-wuerzburg.de)

^1^Rudolf Virchow Center; Center for Integrative and Translational Bioimaging; University of Wuerzburg; Josef-Schneider-Str. 2, Germany, 97080 Wuerzburg, Germany

^2^Technische Universität Berlin; Institute of Chemistry, Straße des 17. Juni 124, 10623 Berlin, Germany

# Supplementary Table

**Supplementary Table 1.** Docking scores for iFSP1 bound to WT FSP1 or to different mutants of FSP1.

| **Mutant** | **Docking score (kcal/mol)** |
| --- | --- |
| WT | -8.9 |
| F21A | -8.6 |
| Y296A | -8.3 |
| F360L/I | -8.1 |
| F21A/F360A | -7.8 |
| F21A/Y296A | -8.1 |
| Y296A/F360A/L | -8.1 |
| F21A/F360A/Y296A | -7.9 |

# Supplementary Figures


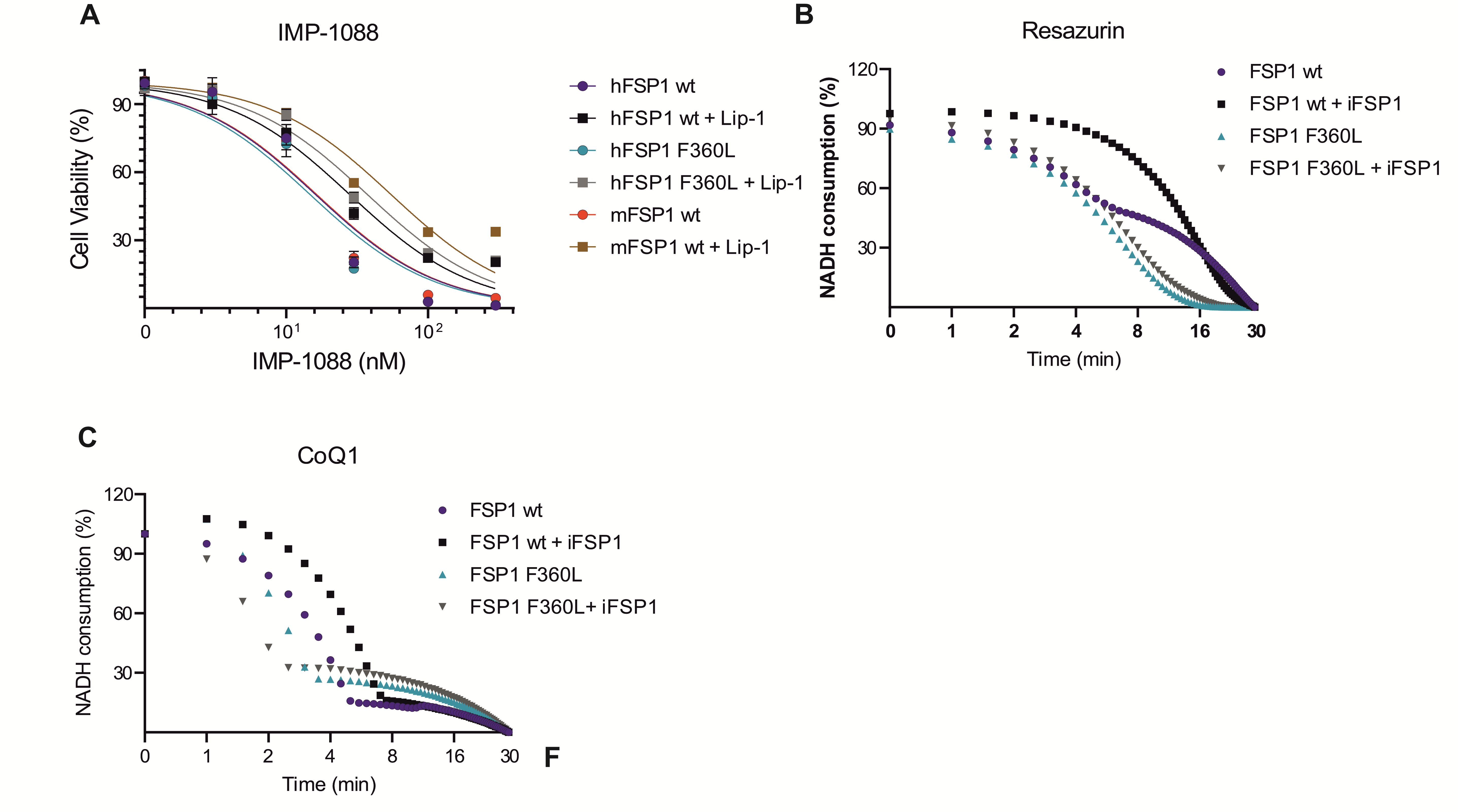


**Supplementary figure 1**: F360L mutation does not interfere in FSP1 activity. (A) Dose-response toxicity to IMP-1088 in Pfa1 *Gpx4*^wt^ overexpressing the hFSP1, mFSP1 and hFSP1^F360L^ mutant with Lip-1. (B-C) NADH consumption assay (340 nm) in TBS buffer using recombinant purified human FSP1 WT and F360L mutant with and without iFSP1, using Resazurin (B) and CoQ1 (C) as substrate. Data represent n = 2 technical replicates of one out of three independent experiments.


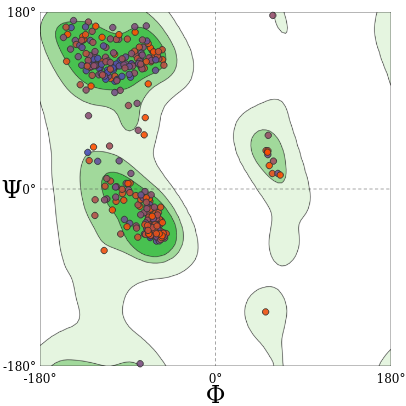


**Supplementary figure 2**: Ramachandran plot for the model of human iFSP1 obtained from AlphaFold2. The structure validation was performed using MolProbity, implemented in the SWISS-MODEL web server. 97.78% of the residues are positioned in favored regions and 0% are in outlier regions. The residues are indicated by dots and colored according to the QMEAN score. The color scale goes from red (bad quality) to blue (good quality).


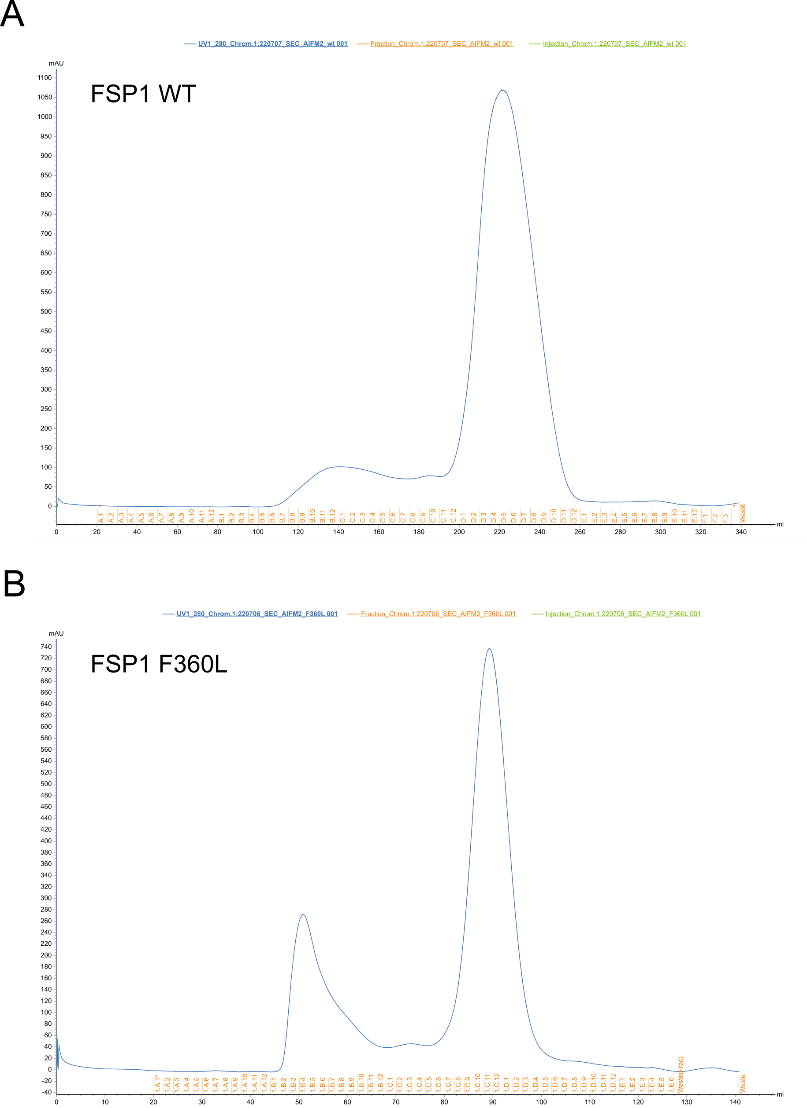


Supplementary figure 3: Size exclusion chromatograms of recombinantly expressed hFSP1 WT and F360L. Pure fractions were collected, pooled, and concentrated.


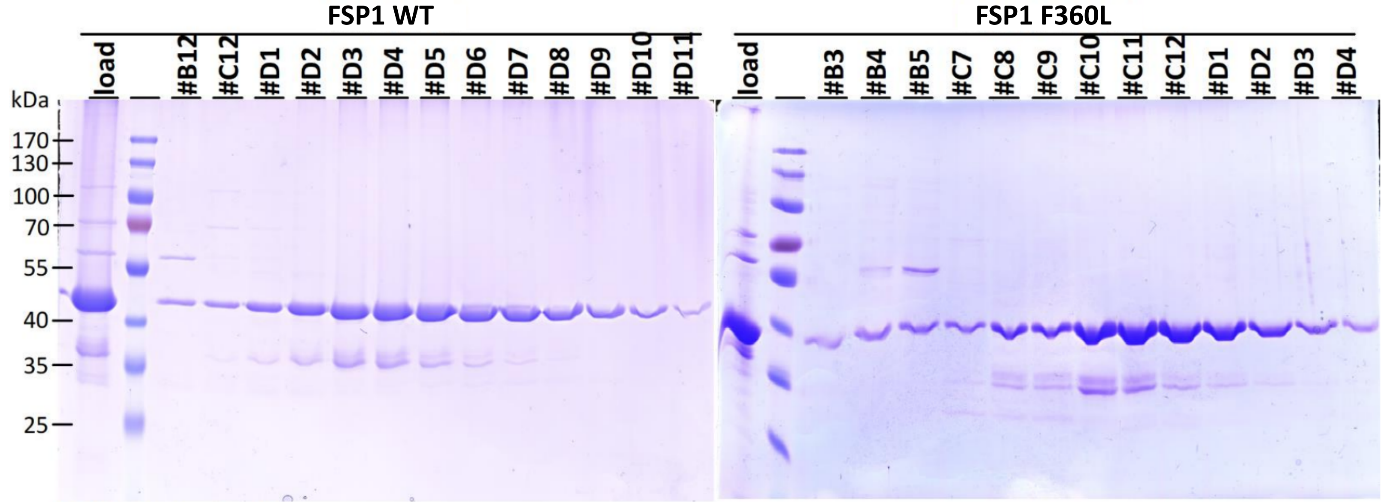


Supplementary figure 4: SDS-PAGE analysis of purified fractions of hFSP1 WT and hFSP1 F360L after size exclusion chromatography. Fractions C12 – D11 were pooled in case of hFSP1 WT and fractions C7 to D4 were pooled in case of hFSP1 F360L.


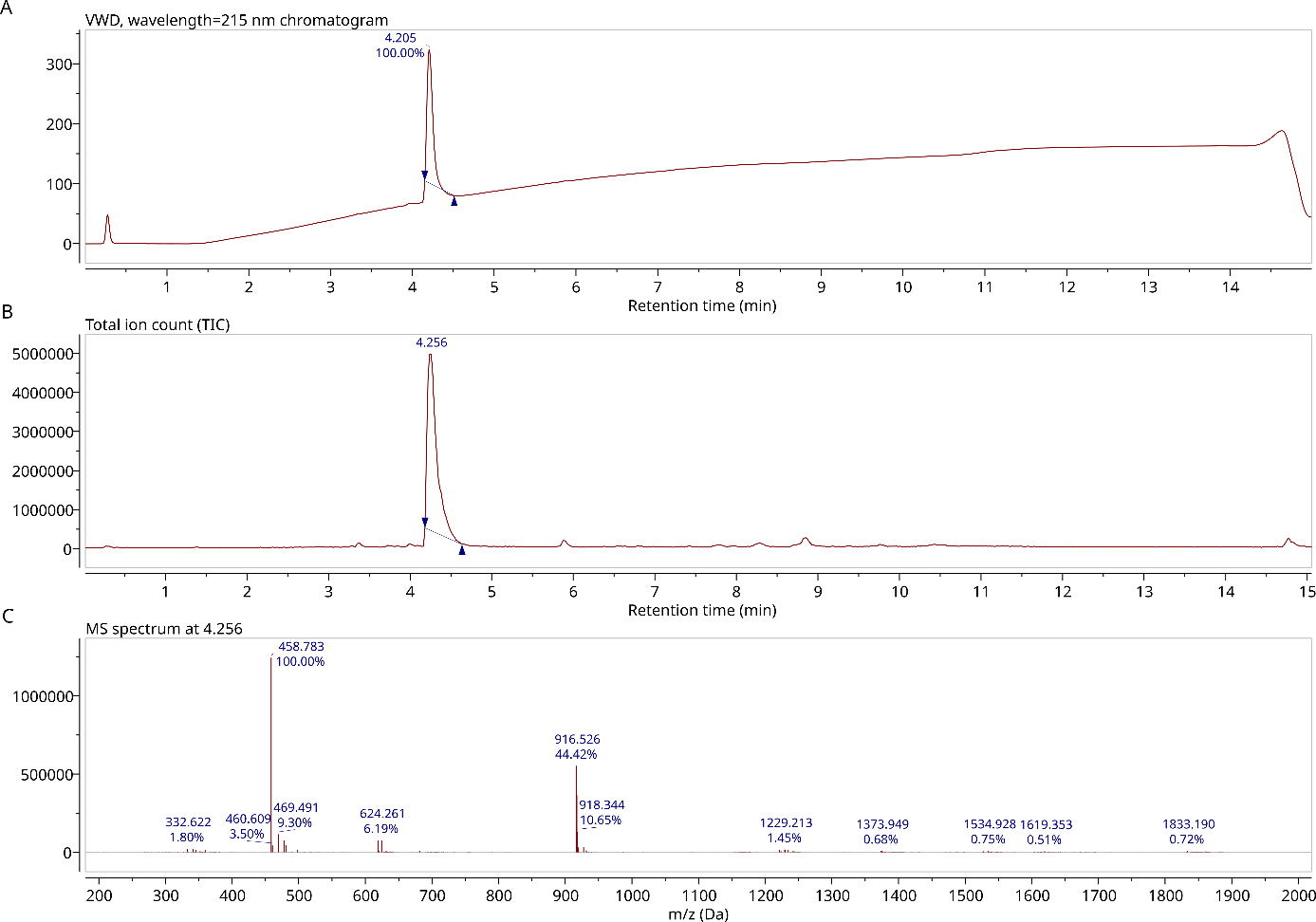


Supplementary figure 5: Purity confirmation of iFSP1-Pip-SCy5 by LC-MS. (A) UV chromatogram at 215 nm wavelength. Note that besides the peak corresponding to the desired product, no other quantifiable peaks were detected. (B) Corresponding total ion count spectrum. (C) MS spectrum corresponding to the main peak identified in UV at 4.2 min. Calculated m/z: 916.35 (+1), 458.68 (+2).
