# Supplementary material for: Molecular characterization of AIFM2/FSP1 inhibition by iFSP1-like molecules
Source: Cell Death Dis. 2023 Apr 21;14(4):281. doi: 10.1038/s41419-023-05787-z (PMC10119282; doi:10.1038/s41419-023-05787-z)

# Supplementary Information

**Molecular characterization of AIFM2/FSP1 inhibition by iFSP1-like molecules**

Thamara Nishida Xavier da Silva^1^; Clemens Schulte^1^; Ariane Nunes Alves^2^, Hans Michael Maric^1^; José Pedro Friedmann Angeli^1^*

*Correspondence to [pedro.angeli@virchow.uni-wuerzburg.de](mailto:pedro.angeli@virchow.uni-wuerzburg.de)

^1^Rudolf Virchow Center; Center for Integrative and Translational Bioimaging; University of Wuerzburg; Josef-Schneider-Str. 2, Germany, 97080 Wuerzburg, Germany

^2^Technische Universität Berlin; Institute of Chemistry, Straße des 17. Juni 124, 10623 Berlin, Germany

Full and uncropped western blot for Figure 1G


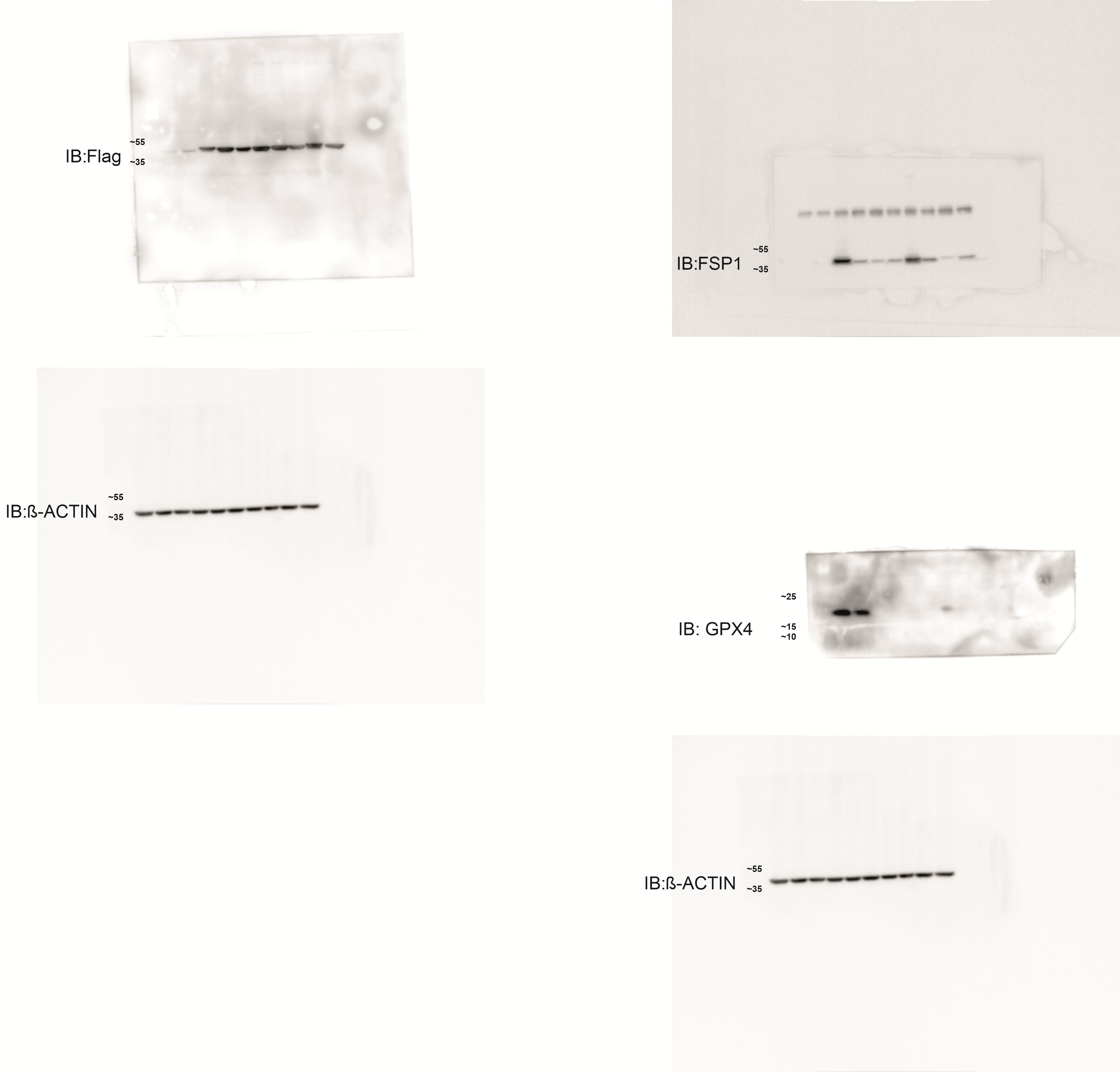


Full and uncropped western blot for Figure 2G


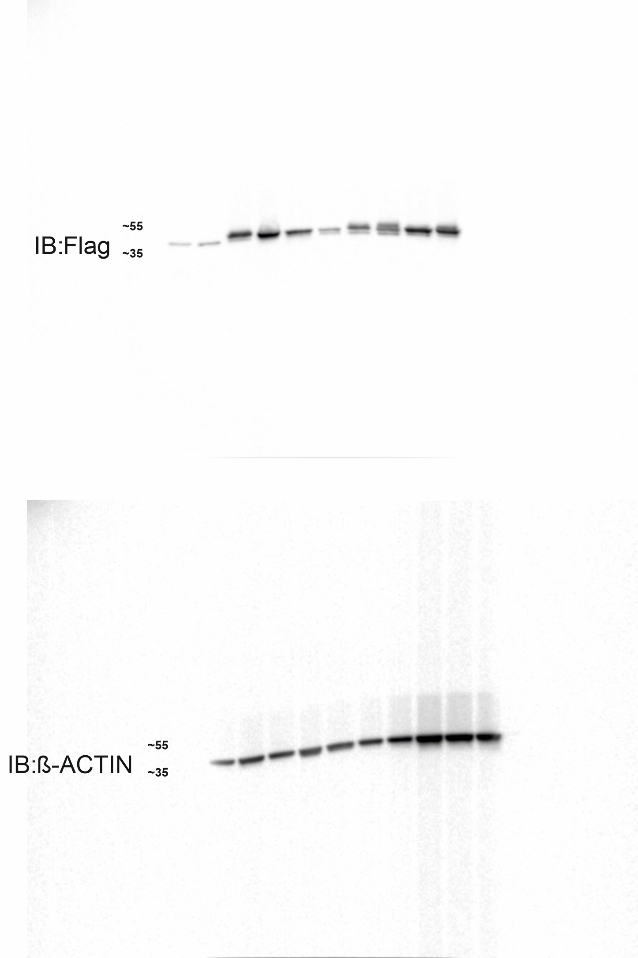

Supplement: Supplementary file 2 — Original Data File [file 41419_2023_5787_MOESM2_ESM.docx]
